# White matter structural connectivity is associated with sensorimotor function in stroke survivors^☆^

**DOI:** 10.1016/j.nicl.2013.05.009

**Published:** 2013-05-27

**Authors:** Benjamin T. Kalinosky, Sheila Schindler-Ivens, Brian D. Schmit

**Affiliations:** a Department of Biomedical Engineering, Marquette University, Milwaukee, WI, USA; b Department of Physical Therapy, Marquette University, Milwaukee, WI, USA

**Keywords:** DTI, diffusion tensor imaging, FA, fractional anisotropy, FOV, field of view, FM, Fugl-Meyer, LDV, log difference volume, LE, lower extremity, MD, mean diffusivity, TE, echo time, TFIRE, Tactful Functional Imaging Research Environment, TR, repetition time, UE, upper extremity, VISC, voxel-wise indirect structural connectivity, Voxel-wise structural connectivity, Tractography, Diffusion tensor imaging, Stroke, Sensorimotor function, Lesion analysis

## Abstract

**Purpose:**

Diffusion tensor imaging (DTI) provides functionally relevant information about white matter structure. Local anatomical connectivity information combined with fractional anisotropy (FA) and mean diffusivity (MD) may predict functional outcomes in stroke survivors. Imaging methods for predicting functional outcomes in stroke survivors are not well established. This work uses DTI to objectively assess the effects of a stroke lesion on white matter structure and sensorimotor function.

**Methods:**

A voxel-based approach is introduced to assess a stroke lesion's global impact on motor function. Anatomical T1-weighted and diffusion tensor images of the brain were acquired for nineteen subjects (10 post-stroke and 9 age-matched controls). A manually selected volume of interest was used to alleviate the effects of stroke lesions on image registration. Images from all subjects were registered to the images of the control subject that was anatomically closest to Talairach space. Each subject's transformed image was uniformly seeded for DTI tractography. Each seed was inversely transformed into the individual subject space, where DTI tractography was conducted and then the results were transformed back to the reference space. A voxel-wise connectivity matrix was constructed from the fibers, which was then used to calculate the number of directly and indirectly connected neighbors of each voxel. A novel voxel-wise indirect structural connectivity (VISC) index was computed as the average number of direct connections to a voxel's indirect neighbors. Voxel-based analyses (VBA) were performed to compare VISC, FA, and MD for the detection of lesion-induced changes in sensorimotor function. For each voxel, a t-value was computed from the differences between each stroke brain and the 9 controls. A series of linear regressions was performed between Fugl-Meyer (FM) assessment scores of sensorimotor impairment and each DTI metric's log number of voxels that differed from the control group.

**Results:**

Correlation between the logarithm of the number of significant voxels in the ipsilesional hemisphere and total Fugl-Meyer score was moderate for MD (R2 = 0.512), and greater for VISC (R2 = 0.796) and FA (R2 = 0.674). The slopes of FA (p = 0.0036), VISC (p = 0.0005), and MD (p = 0.0199) versus the total FM score were significant. However, these correlations were driven by the upper extremity motor component of the FM score (VISC: R2 = 0.879) with little influence of the lower extremity motor component (FA: R2 = 0.177).

**Conclusion:**

The results suggest that a voxel-wise metric based on DTI tractography can predict upper extremity sensorimotor function of stroke survivors, and that supraspinal intraconnectivity may have a less dominant role in lower extremity function.

## Highlights

• An intrinsic voxel-based structural connectivity metric is proposed.
• The metric enhances the impact of stroke lesions on the distant voxels.
• Whole-brain extralesional anatomical connectivity predicts functional outcome.
• Functional impact of a lesion is determined by residual anatomical connectivity.
• Connectivity to the posterior parietal cortex is a key to sensorimotor function.

## 1 Introduction

Diffusion tensor imaging (DTI) of brain white matter structural connectivity may have prognostic value for acute stroke patients at risk of motor impairment. In particular, DTI of the corticospinal tract has been a primary focus for predicting stroke severity and clinical outcome (Puig et al., 2010; Thomalla et al., 2004). In the corticospinal tract of stroke survivors, DTI measures that indicate structural integrity in white matter correlate with muscle strength (Chen et al., 2008; Puig et al., 2010; Schulz et al., 2012), walking ability (Jayaram et al., 2012), hand function and motor recovery (Lindenberg et al., 2010; Schaechter and Fricker, 2009; Thomalla et al., 2004; Vargas et al., 2012). Corticospinal tract size and damage to the corticospinal tract, estimated using DTI in the acute setting, also correlate with long-term recovery (Pannek et al., 2009; Parmar et al., 2006; Zhu et al., 2010). In addition to the natural recovery from stroke, information about corticospinal tract loss predicts the extent of motor recovery obtained from therapeutic interventions (Riley et al., 2011; Stinear et al., 2007). Thus, the predominant approach in developing imaging biomarkers in stroke survivors has been corticospinal tract-specific measures based on manually-identified or atlas-based regions of interest (Borich et al., 2012). These previous approaches highlight the potential value in utilizing DTI data to predict functional outcomes; however, analyses based on specific regions of interest require subjective region selection, and might not account for impairments associated with damage to or connections to other regions of the brain. The purpose of the current study was to develop and test a new imaging parameter as a biomarker for sensorimotor function in stroke survivors based on a whole brain, voxel-wise analysis of anatomical connectivity.

Although DTI measures of the corticospinal tract provide valuable information about stroke, a whole brain voxel-based analysis of brain structure might have advantages over corticospinal tract region of interest approaches. Namely, voxel-based analyses are simple to apply, objective, and test the structural changes across the entire brain. A voxel-based analysis involves the normalization of images (through registration and spatial filtering) followed by statistical comparisons of DTI parameters of the resulting maps (Ashburner and Friston, 2000; Wright et al., 1995). These analyses have been applied to DTI parameters of the brain in normal development and aging (Della Nave et al., 2007; Snook et al., 2007), following traumatic injury (Bendlin et al., 2008; Chu et al., 2010) and during progressive disease (Agosta et al., 2007; Sage et al., 2009; Thivard et al., 2007). Conversely, there are limitations to voxel-based analyses including dependence on the quality of image registration across subjects and effects of smoothing applied to the images (Abe et al., 2010; Ashburner and Friston, 2001; Bookstein, 2001; Van Hecke et al., 2011). Consequently, an alternative voxel-based approach for assessing brain white matter, Tract-Based Spatial Statistics (TBSS) (implemented within the FMRIB Software Library (FSL)) has been developed (Smith et al., 2006). This technique accounts for the registration and smoothing issues by using a tract ‘skeleton’ obtained from fractional anisotropy (FA) values. In addition to a number of other applications, TBSS has been applied to the brain of stroke survivors and detects FA changes in white matter tracts that correlate to upper extremity function (Schaechter and Fricker, 2009).

Incorporating measurements of white matter structural connectivity of the brain within DTI voxel-based approaches may offer additional opportunities for the characterization of structural changes after stroke. The loss of white matter tracts after stroke has implications throughout the brain, including functional processes that require the integration of information from multiple brain areas. The primary tool for characterizing the structural connectivity between brain regions is DTI tractography (Conturo and Lori, 1999; Jones and Simmons, 1999; Mori et al., 1999). Tractography models have been used to identify anatomical tracts and features of the tractography analysis, such as the number of fibers passing through a voxel (Calamante et al., 2010; Roberts et al., 2005). A structural connectivity matrix can then be obtained by combining white matter fiber trajectories with gray matter anatomical regions of interest segmented from a high resolution anatomical MR image (Hagmann et al., 2007; Sporns, 2011). This matrix represents the anatomical connectivity of the specific regions of the brain, but depends on the segmentation of specific regions of gray matter as nodes in the connectivity matrix. In contrast, voxel-based connectivity models make no assumptions about the parcellation of brain volume into ROIs, nor do they require a priori knowledge about the physiology of the tissue within a voxel (Scheinost et al., 2012). The absence of assumptions in a voxel-based approach is appealing for generalizing connectivity models for clinical application.

In this study, we developed a unique metric of structural connectivity as a biomarker for loss of sensorimotor function in subjects with chronic stroke. Our metric characterized the anatomical connectivity of each voxel of the brain based on diffusion tractography (i.e. a voxel-wise indirect structural connectivity (VISC)). This VISC metric was designed to have high sensitivity to lesions of prominent white matter tracts, which normally connect large numbers of voxels. A voxel-based analysis of stroke and control brains was conducted on the VISC metric and compared to a voxel-based analysis of FA and mean diffusivity in the same samples. Sensitivity to sensorimotor function was tested by correlating the volume of differences in VISC, between stroke subjects and controls, with sensorimotor impairment measured by the Fugl-Meyer Assessment (Fugl-Meyer, 1975).

## 2 Methods

### 2.1 Data collection

#### 2.1.1 Subject recruitment and Fugl-Meyer testing

Ten subjects with chronic post-stroke hemiparesis (5 female, age 55.20 ± 7.06 years, at least 1.1 years since stroke) and nine age-matched control subjects (6 female, age 53.40 ± 13.10 years) participated in this study. Each subject provided written consent to the experimental protocol, which was approved by the Institutional Review Boards at Marquette University and the Medical College of Wisconsin. In recruiting subjects, a sample of convenience was used. General inclusion criteria were ability to provide informed consent and the ability to move the legs with no contraindications to light exercise. Additional inclusion criteria for stroke survivors were a single cortical or subcortical stroke at least 6 months earlier, clinically detectable movement impairment on one side of the body, communication adequate to follow instructions for the experiment, and no neurological impairments other than stroke. Control subjects had to be free of stroke or other neurological impairments.

Each stroke subject completed a slightly modified system of upper extremity (UE) and lower extremity (LE) portions of the Fugl-Meyer (FM) Assessment (Fugl-Meyer, 1975) for global impairment (maximum possible score is 130 for UE and 96 for LE). The scoring system for the FM Assessment is shown in Table 1, and the FM scores for each subject are shown in Table 2. Note that lower scores indicate greater impairment. FM assessments were completed by a physical therapist with 9 years of clinical experience. Reliability and validity assessments were not done for this study; however, the FM has been shown to have excellent construct validity, good concurrent validity with other stroke motor scores, satisfactory predictive validity for functional level at discharge from hospital (r = 0.72), and excellent intra- and inter-tester reliability (ICC = 0.98) (Gladstone et al., 2002; Hsueh et al., 2008). The maximum score for the UE portion of FM is 130 because it includes UE reflexes (max = 6), UE movements in and out of synergy (max = 30), voluntary movements of the wrist and hand (max = 24), and UE coordination (max = 6), parachute responses (max = 4), UE light touch (max = 4), UE proprioception (max = 8), UE range of motion (max = 24), and UE pain (max = 24). Nevertheless, the scale required adjustment to better reflect the possible range of scores. Since the control subjects did not have any lesions and the Fugl-Meyer assessment is a measure of impairment, sensorimotor function in control subjects was not tested.

#### 2.1.2 MRI scans

After completing MRI safety screening, the nineteen subjects were imaged with a 3 T clinical MR system (GE Signa Excite, GE Healthcare, Milwaukee). For each subject, an axial DTI sequence was acquired with one b0 image, 25 noncollinear, equally spaced diffusion directions, b-value = 1000 s/mm^2^, matrix = 128 × 128, FOV = 24 cm, slice thickness = 4 mm, TE = 86.5 ms, TR = 10 s, NEX = 2. As an anatomical reference, a sagittal T1-weighted image with 1 mm isotropic resolution was acquired using a spoiled gradient recalled (SPGR) pulse sequence.

### 2.2 Subject-specific data processing

#### 2.2.1 Diffusion tensor calculation

From the diffusion weighted images acquired for each subject, twenty-five diffusion coefficients were calculated at each voxel as the signal loss between the diffusion-weighted signal and zero-diffusion signal. These diffusion coefficients were fit to a second order tensor model by the least squares method. The diffusion tensor matrix was then diagonalized to derive three eigenvalues and eigenvectors. The three eigenvalues (*λ*_1_,*λ*_2_,*λ*_3_) were used to calculate the MD and FA (Basser et al., 1994).

#### 2.2.2 Image registration

**Fig. 1:**
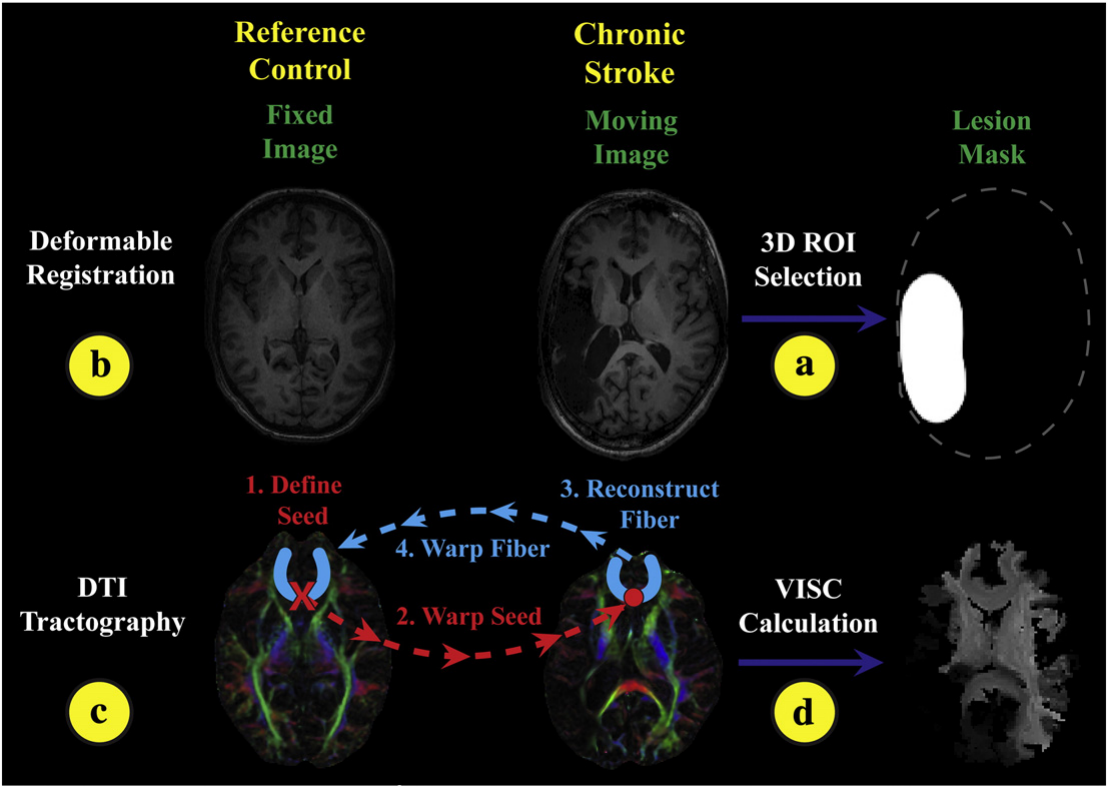
Overview diagram of methods for image registration, DTI tractography, and VISC calculation. (a) A lesion mask was manually selected from the stroke subject's T1-weighted image, and then (b) the stroke subject was registered to the reference control subject. (c) The registration transform was used to warp seeds from the reference control space to perform DTI tractography in the stroke subject's space. Reconstructed fibers were then transformed back into the reference control space and (d) used to perform structural connectivity analysis. Furthermore, a novel voxel-wise indirect structural connectivity (VISC) metric was calculated. Control subjects were processed similarly except without lesion correction.

Anatomical brain images from all control and stroke subjects were registered to a reference image (Fig. 1b) in order to compare DTI metrics between individual stroke subjects and the control group. The control subject with characteristics closest to Talairach space was selected as the reference. Each subject's T1-weighted image was then registered to the reference subject using a deformable image registration framework implemented in Tactful Functional Imaging Research Environment (TFIRE, http://www.eng.mu.edu/inerl/tfire), an in-house Java-based software platform. A nine-parameter affine registration was performed before proceeding with Thirion's demons deformable image registration method (Thirion, 1998). The output from this process was a 3D displacement field that mapped each voxel center in the fixed reference control space to a physical location in the subject space.

##### 2.2.2.1 Stroke lesion selection and correction

**Fig. 2:**
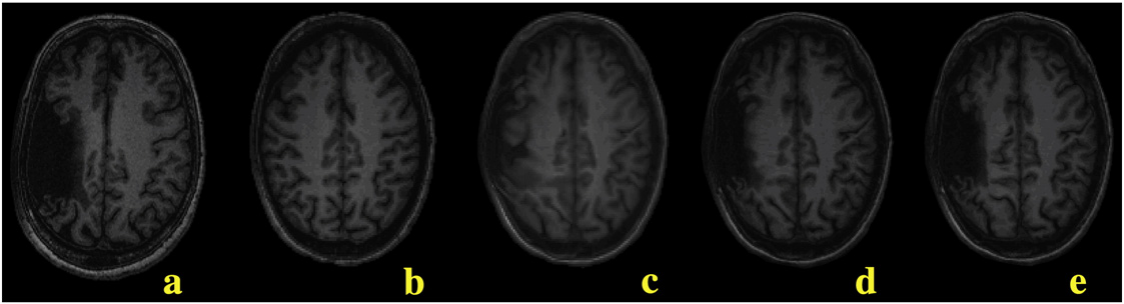
In registering a stroke brain (a) to the template control (b), the original deformation algorithm (c) was modified to include lesion masking (d) and anisotropic smoothing (e) to produce the best result.

Initial registration results for stroke subjects were determined unacceptable based on visual observation. In particular, areas in the control image were mapped to the lesion boundary of the stroke subject. Lesion masking and a modified smoothness constraint were introduced to preserve anatomical features near the lesion boundary (Fig. 2). A lesion mask was manually identified for all slices using a custom user-interface. Although some lesions were discontinuous in some slices, they were not broken up volumetrically. Only one contiguous unilateral lesion was selected for each subject. During the registration, displacements that mapped the fixed image to locations inside the lesion mask region were not updated. An edge-preserving Gaussian filter was used to impose piece-wise continuity. This modified filtering allowed tissue surrounding the lesion to register, while preserving the lesion features.

##### 2.2.2.2 Inverse deformation estimate

Aside from challenges with stroke lesion mapping, the deformable registration was performed in a small deformations setting, which led to a non-invertible deformation field. However, an inverse transform was needed for our subsequent tractography approach. We mitigated this problem by approximating an inverse with the following method. First, a 3D displacement field was initialized to zero at each location. The forward transform was used to map each physical coordinate in the fixed subject image space to a location in the reference (control) image space. The displacement of the inverse transform at this location was forced to map back to the fixed image coordinate. Since this adjustment resulted in inhomogeneous mappings in the fixed image space, the inverse transform was smoothed with a 2 mm full-width half-max Gaussian filter. The adjustment and smoothing steps were repeated for 10 iterations. This iteration number was chosen heuristically to balance the tradeoff between computation time and residual error. The mean and standard deviation of the final error magnitude across subjects was 0.5622 ± 0.3326 mm. This process produced forward and inverse transformations between each subject and the reference control based on the anatomical (T1 weighted) images. These transformations were subsequently used for the diffusion image data.

##### 2.2.2.3 Diffusion to anatomical MRI

In order to use the anatomical image transformations for aligning the subjects in DTI space, each subject's T1-weighted image was registered to his or her FA image. The SPGR and FA images were histogram-matched, and then a nine-parameter 3-dimensional affine transformation was optimized using a gradient descent algorithm with a mean squared difference cost function.

#### 2.2.3 Tractography and voxel-wise indirect structural connectivity metric

##### 2.2.3.1 DTI tractography

Our in-house software, TFIRE, was also used for DTI tractography (Fig. 1c) and structural connectivity analysis (Fig. 1d). We chose to define of a voxel by the location of its center. This convention allowed for a straight-forward one-to-one mapping between a connectivity matrix and an image space. Further detail is provided in the Appendix A.

Using the reference control DTI data, tractography was initialized by seeding with uniform 1 mm spacing in all areas with an FA above 0.3. The coordinate of each seed was transformed from control into subject space. The FACT method (Mori et al., 1999) was implemented in TFIRE and used to reconstruct fiber trajectories seeded at each voxel center. Specifically, the principle eigenvector with an FA magnitude was integrated using a 4th order Runge–Kutta technique with a step size of 0.1 mm. A maximum angle of curvature of 60° was used to terminate propagation at voxels with crossing white matter fiber bundles. Since the endpoints of fibers often converge at gray matter voxels, a minimum FA stopping criterion of 0.15 was used to allow the fibers to propagate into the gray matter. Including white matter voxels in the VISC calculation was intended to account for bifurcating and converging fiber pathways. After construction was completed, all fibers with length < 1 cm or > 14 cm were excluded. Each reconstructed white matter fiber was transformed back into the reference control space using the inverse registration transform.

##### 2.2.3.2 Theoretical framework

**Fig. 3:**
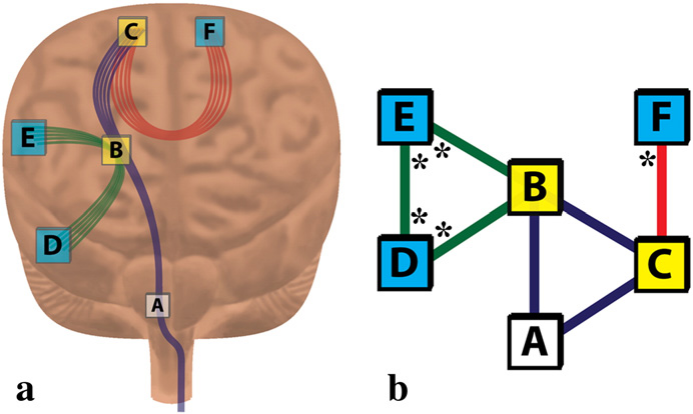
Example calculation for the voxel-wise indirect structural connectivity (VISC). The “anatomical” diagram (a) and the theoretically equivalent network graph (b) provide the information necessary to calculate the VISC of voxel A. Voxels B and C (yellow) are directly connected to voxel A by at least one common fiber. Voxels D, E, and F (aqua) are indirect neighbors to voxel A because they are not directly connected to A by any fiber but do share a common direct neighbor (B or C) with A. The VISC of voxel A is the average number of direct connections to its indirect neighbors. These direct connections are marked with an * in (b). Since there are 5 total direct connections for its 3 indirect neighbors, the VISC of voxel A is 5/3.

In order to model the effects of a stroke lesion at the voxel-level, a measure of voxel-wise indirect structural connectivity (VISC) was developed. The desired properties of VISC were to reproducibly amplify lesion-induced effects on white matter structural connectivity of the entire brain, and to intrinsically measure these effects at the voxel-level. We started by defining the structural connectivity of the whole brain based on the voxel-wise direct and indirect connections obtained from DTI tractography (Fig. 3). As an example, consider an axonal fiber pathway that structurally connects three voxels; A, B, and C in Fig. 3. We defined B and C to be direct neighbors to A by their structural connection. There are also axonal pathways that connect B or C to voxels other than A. These pathways indirectly connect A to voxels D, E, and F, which we define as indirect neighbors of voxel A. The connectivity graph for this example is shown in Fig. 3b. The VISC of voxel A is the average number of connections to indirect neighbors D, E, and F. Since F has one direct connection, and D and E each have two direct connections, the VISC of A is 5/3.

##### 2.2.3.3 Voxel-wise indirect structural connectivity (VISC) calculation

As a foundation for deriving a voxel-wise connectivity metric, the reconstructed fiber trajectories were expressed as sets of coordinates in the template control subject's DTI image-space. Binary matrix $\tilde{X}$ represents the direct connectivity between the voxels penetrated by one reconstructed fiber, where ${\tilde{x}}_{\mathrm{ij}}$ is 1 if the *i*th and *j*th voxels are both penetrated by that fiber and 0 otherwise. Matrix **X** represents the direct connectivity between all voxels in an image, where *x*_*ij*_ is 1 if the *i*th and *j*th voxels are both penetrated by at least one fiber and 0 otherwise. Thus, **X** is the union of individual $\tilde{X}$ across all fibers. Directly calculated from **X**, matrix **Y** represents the indirect connectivity between all voxels in an image, where *y*_*ij*_ is 1 if the *i*th and *j*th voxels share a directly connected voxel but are not directly connected to one another. Then row vector **y**_(*i*)_ from **Y** represents the indirect connections of the *i*th voxel. Using **1** as the summation vector, the VISC of the *i*th voxel in an image is its total number of direct connections to its indirect neighbors (expressed as **y**_(*i*)_**X1**) divided by its total number of indirect neighbors (expressed as **y**_(*i*)_**1**).

(1) $${\mathrm{VISC}}_{i}=\frac{y_{\left(i\right)}X1}{y_{\left(i\right)}1}=\frac{\sum_{j}^{N}\left(y_{\mathrm{ij}}\sum_{k}^{N}x_{\mathrm{jk}}\right)}{\sum_{j}^{N}y_{\mathrm{ij}}}$$

Previously introduced voxel-wise metrics based on DTI tractography, such as fiber count and mean fiber length (Roberts et al., 2005), are correlated with FA. In order to consider whether fiber count information affected the correlation of VISC with FA, we incorporated a connection count weighting factor *α*. This contrast mechanism gives weight to the total number of connections to a voxel's indirect neighbors. As *α* is decreased from 1 to 0, the VISC approximates the *total number* rather than the *mean number* of direct connections to a voxel's indirect neighbors, with VISC parameterized by *α* as

(2) $$\mathrm{VISC}{\left(\alpha \right)}_{i}=\frac{y_{\left(i\right)}X1}{{\left(y_{\left(i\right)}1\right)}^{\alpha }}\text{.}$$

#### 2.2.4 Effect of tractography parameters and contrast parameter *α* on VISC

Parameters used for tractography and VISC connection weighting were manipulated to determine their effect on the final VISC value. Since VISC is derived from DTI tractography, it is expected to depend on the stopping criteria for fiber propagation. We considered that VISC might decrease as FA threshold increased due to a more conservative stopping criterion. If the minimum FA is increased, then propagation ends more readily and reconstructed fibers are shortened. Thus, the volume occupied by reconstructed white matter fibers decreases as FA threshold increases. Since VISC is based on structural connectivity with surrounding and distant voxels, it can also be interpreted as a volume-based metric. If the volume occupied by fibers decreases proportionally with increased FA threshold, then the percent change in volume is higher at lower FA thresholds. Thus, the logarithm of the volume of VISC was expected to be negatively correlated with the FA threshold. To test this hypothesis, we calculated the mean VISC of 3 voxels selected from the center of each of the cerebral peduncles, which are known to have a high white matter fiber density. The purpose of selecting only 3 voxels was to understand how the VISC in ipsilesional voxels and their contralesional homolog were affected by FA threshold in an anatomical region with high fiber density. We manipulated the FA threshold from 0.10 to 0.40 in increments of 0.05, and the mean VISC of the cerebral peduncle ROI was calculated for each FA threshold setting. Correlation analyses were performed between the mean VISC and FA threshold. The analysis was repeated for the left and right ROIs of the control subjects, and the ipsilesional and contralesional ROIs of the stroke subjects. A whole-brain analysis was also performed in which the VISC and FA threshold were correlated across all voxels with nonzero VISC in the reference control brain.

Since high FA may be an indicator of anatomical white matter fiber density, a voxel-wise metric based on DTI tractography might correlate with FA. For example, the fiber density index (FDi) introduced by Roberts et al. (2005) represents the number of fibers penetrating a voxel and was found to be correlated with FA. In every subject, the FA and VISC were regressed across all voxels in the reference space. The mean and standard deviation of R^2^ values were plotted. The R^2^ values with FA were also plotted for mean fiber length and fiber count for visual reference.

We also considered that the total number of connections to a voxel might influence VISC or its correlation to FA. If the number of structural connections to a voxel increases with greater FA, then it is possible that greater connection number weighting (decreasing parameter *α*) might increase the correlation between FA and VISC. The direct effect of total connections on VISC was qualitatively analyzed by manipulating *α* from 1 to 0 and visualizing the changes in the contrast of the reference control's VISC image. Additionally, the whole-brain correlation analysis between FA and VISC was repeated for each setting of VISC.

#### 2.2.5 Statistical analyses

##### 2.2.5.1 Whole-brain analysis

A whole brain voxel-based analysis was used to compare each stroke subject to the controls. For each voxel, a one-tailed Student's *t*-test was performed on the differences of the 9 controls with a single stroke subject, with a zero mean difference null hypothesis. The resulting number of significant voxels, or difference volume, was considered as a possible predictor of Fugl-Meyer score. It can also be interpreted as the percent of effected brain volume. As the difference volume becomes large, a further increase leads to a lower percent change in volume. Consequently, we expected that increasing the size of a small difference volume would lead to a greater percent change in impact on function. Thus, we hypothesized that the log number of significant voxels, or logarithmic difference volume (LDV), would increase as Fugl-Meyer score decreased. A simple linear regression was performed for each DTI metric, with its LDV as a predictor of Fugl-Meyer score in stroke subjects. Separate regressions were conducted for FA, VISC, and MD using Matlab (R2009b, The Mathworks, Natick, MA). For each regression, an F-test was used to test the slope for significance. In a second set of simple linear regressions, the mean FA and VISC in the ipsilesional hemisphere were regressed against total FM score. In order to prevent voxels with zero VISC from contaminating the mean VISC, the means were first calculated for only voxels with a nonzero VISC. However, the regression was also repeated with all ipsilesional voxels included. In summary, nine simple linear regressions were performed in the whole-brain analysis, with three tests per voxel-based measure.

Four multiple linear regressions were performed to determine the unique relationships between the differences in DTI measures and sensorimotor impairments. The Fugl-Meyer score was divided into subcomponents with two strategies. The first approach split the FM score into its five domains; motor (100 max), balance (14 max), sensation (24 max), range of motion (44 max), and pain (44 max). The second approach divided the total FM into two groups: upper extremity (130 max) and lower extremity (96 max). The whole-brain LDVs of VISC, FA, and MD were regressed as predictors of each set of FM subscores. Two more multiple linear regressions were performed, with the *mean* VISC, FA, and MD in the ipsilesional hemisphere as the independent variables. In all multiple regression tests used in this study, multiple comparisons correction was performed by dividing the Type I Error rate, α, by the number of metrics, being 3.

Although the VISC metric was developed for the purpose of enhancing detection of the effects of brain lesions, the unknown variability of VISC in the control group could compromise the calculation of the difference volume. The intersubject reproducibility of VISC within the control group was tested by calculating the whole-brain LDV between each control subject and the other controls. We found zero significantly different voxels in every control subject. Lesion selection could also affect the reproducibility of VISC in an image. A second investigator reselected all lesions, and then image registration and analyses were repeated. Across all voxels and subjects, the percent difference in VISC due to lesion selection was 1.52 ± 0.37%. Lesion-selection did not change the findings in this study.

##### 2.2.5.2 Region-based *post hoc* analysis

Since we expected the logarithmic difference volume (LDV) of each DTI metric within specific brain regions to reflect its volume of effect, a region-based correlation analysis was performed. Using the same registration algorithm performed on the T1-weighted images, the fractional anisotropy image of the template control was registered to the Johns Hopkins University “Eve” atlas (Oishi et al., 2009) in Talairach coordinates, also named JHU-Talairach-ss in MRIStudio. The registration transform was used to warp 85 ipsilesional regions of interest (ROIs) from the atlas space into the reference control space. For each ipsilesional ROI, the same set of simple linear regressions used in the whole-brain analysis was repeated.

If the total number of significantly different voxels in a particular region were correlated with Fugl-Meyer, then the raw measurement itself may share that correlation. To address this question, linear regressions were performed with the *mean* VISC, FA, and MD of every ipsilesional ROI in stroke subjects as a predictor of FM score. As with the regressions involving the log difference volumes, an F-test was used to test each slope for significance. In comparison to mean FA and MD, we expected that the mean VISC would correlate with FM score in an additional set of effected regions distant from a lesion.

## 3 Results

### 3.1 Comparison of VISC, fiber count, and mean fiber length

**Fig. 4:**
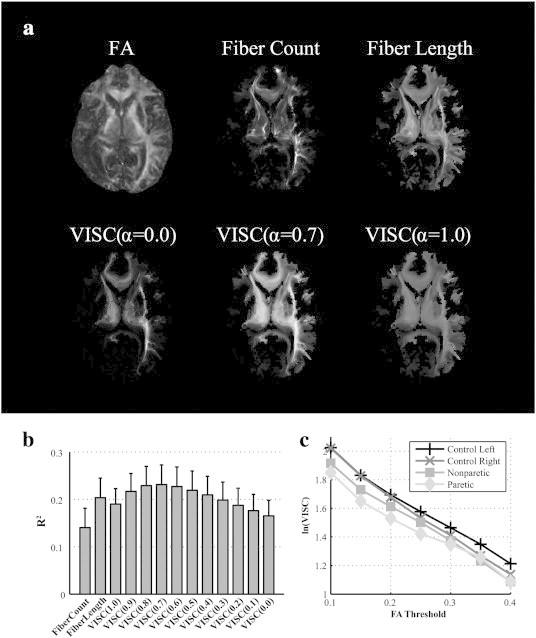
Voxel-wise indirect structural connectivity (VISC) correlations and contrasts. (a) Visual comparison of VISC, fiber count, and mean fiber length for a central axial slice of a stroke subject at the level of the internal capsule. The indirect connectivity weighting factor, *α*, was manipulated from 0 to 1 to observe its effect on the spatial contrast of VISC. (b) The R^2^ values of FA with several tractography-derived measures. The VISC at different settings of *α* are labeled as VISC(*α*) (e.g. VISC(1.0) for *α* = 1.0). The mean and standard deviation in R^2^ values across all 19 subjects included in this study are shown. (c) The natural log of the mean VISC in the cerebral peduncle is plotted against the minimum FA threshold used as a stopping criterion for DTI tractography. The same bilateral ROIs were used to report this relationship in the paretic/ipsilesional (diamond) and nonparetic/contralesional (square) hemispheres of stroke subjects, and the left (+) and right (x) hemispheres in control subjects.

VISC was compared with fiber count and mean fiber length by adjusting parameter *α* (Fig. 4a, b). Fiber count was calculated as the number of fibers penetrating a voxel, and mean fiber length was calculated as the average physical length of these fibers. These two measures were sensitive to the number of degenerate fibers passing through a voxel, which led to undesired local hyperintensities. If *α* was set to zero in Eq. (2), then the denominator of VISC became 1, which equated VISC with its numerator, **y**_(*i*)_**X1**. In this case, VISC was greatest in voxels with a high calculated fiber count and number of direct connections. As *α* was increased from 0 to 0.7, the VISC metric provided an enhanced contrast similar to mean fiber length. This similarity was a consequence of including all white matter voxels in the VISC calculation. Setting *α* = 1 was determined to minimize inter-subject variability between controls. Although *α* was an important parameter in developing the VISC metric, Eq. (1) was used to calculate VISC in all group analyses.

### 3.2 Weak correlations of FA with VISC, mean fiber length, and fiber count

In order to compare the correlations of FA with multiple DTI tractography-derived metrics, the coefficient of determination between each metric and FA was calculated in each subject across all voxels with an FA above 0.15 (a stopping criterion during tractography). These metrics included mean fiber length, fiber count, and VISC at 11 different settings of *α* from 0.0 to 1.0 (Fig. 4b). The R^2^ values of individual subjects are listed in Table 3. Subject sample mean and standard deviation of R^2^ values were calculated for each metric. The correlation of FA with VISC (α = 1) suggested a small but significant portion of VISC variability can be predicted by the FA (R^2^ = 0.184 ± 0.033). As a point of reference, the correlations between FA and mean fiber length (R^2^ = 0.199 ± 0.041), and fiber count (R^2^ = 0.140 ± 0.041) were comparable. As *α* was decreased from 1.0 to 0.0, the correlation between VISC and FA increased until *α* = 0.7, which then *decreased* as *α* was further adjusted 0.7 to 0.0.

### 3.3 Tractography minimum FA stopping criterion correlates with VISC

As expected, the mean VISC decreased exponentially as minimum FA threshold (tractography stopping criterion) increased. The logarithm of the mean VISC metric was strongly correlated (R^2^ = 0.991 ± 0.007) with tractography FA threshold across subjects. The log mean contralesional and ipsilesional VISC were both lower than controls for all FA thresholds (Fig. 4c); however, FA thresholds less than 0.15 led to the greatest contrast between control and stroke VISC. At greater FA thresholds, the difference in VISC between the left and right cerebral peduncles increased in control subjects and decreased in stroke subjects. Although we selected an FA threshold of 0.15 in correspondence to past literature (e.g. An FA threshold of 0.18 was used in (Chen et al., 2008)), our results suggested that this threshold was also appropriate for contrasting controls with chronic stroke subjects. Across all voxels in the normalized space, the correlation between VISC and FA threshold was R = − 0.950 ± 0.044, and the correlation between log(VISC) vs. FA threshold was R = − 0.978 ± 0.025.

### 3.4 VISC highlighted brain areas distant from the lesion

**Fig. 5:**
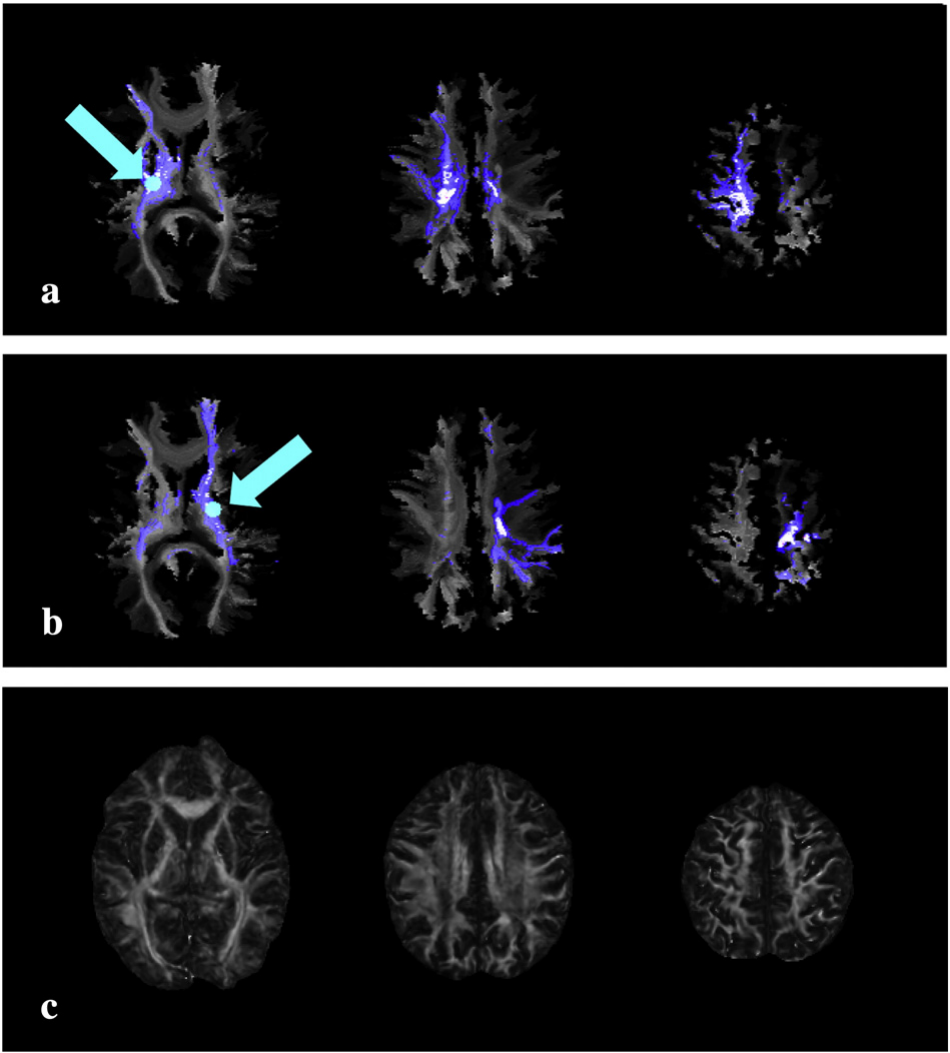
Bilateral voxels were selected from the posterior limb of the internal capsule (PLIC) in a chronic stroke subject with a lesion localized to the right anterior limb of the internal capsule (ALIC). Overlaid on lesioned (panel a) and non-lesioned (panel b) sides of the subject's VISC image, the direct neighbors (white) and their direct connections (blue) of each selected voxel (cyan) encompass the sensorimotor area in the most superior slice. Although the fractional anisotropy (panel c) in the cortex of the lesioned hemisphere was similar to the contralesional hemisphere, the VISC (panels a and b) was reduced due to the ALIC lesion.

**Fig. 6:**
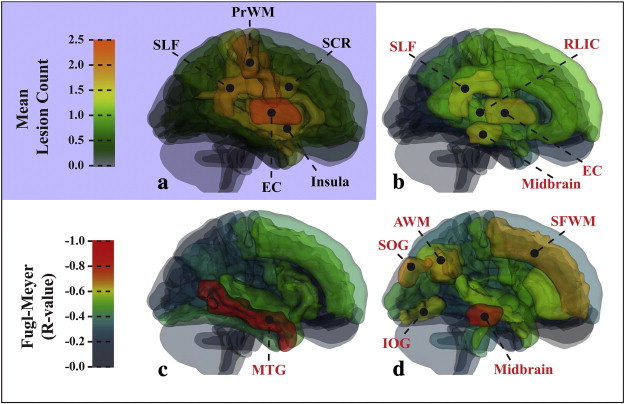
Diagram of ipsilesional ROIs in the control template space, showing the lesion distribution and the correlation coefficients of logarithmic difference volume and composite Fugl-Meyer (FM) score. (a) The lesion distribution of the stroke group was most dense in the EC, SLF, PrWM, SCR, and Insula. (b) The MD log difference volume and FM score were significantly correlated (p < 0.05) in the EC, midbrain, SLF, and RLIC. (c) Correlations with total FM score were significant (p < 0.05) for FA log difference volume in the MTG. (d) The VISC log difference volume was correlated (p < 0.05) with FM score in the midbrain, SFWM, AWM, SOG, and IOG. Acronyms: angular gyrus white matter (AWM); external capsule (EC); middle temporal gyrus (MTG); precentral gyrus white matter (PrWM); superior corona radiata (SCR); superior frontal gyrus (SFG); superior frontal white matter (SFWM); superior longitudinal fasciculus (SLF); superior temporal gyrus (STG).

**Fig. 7:**
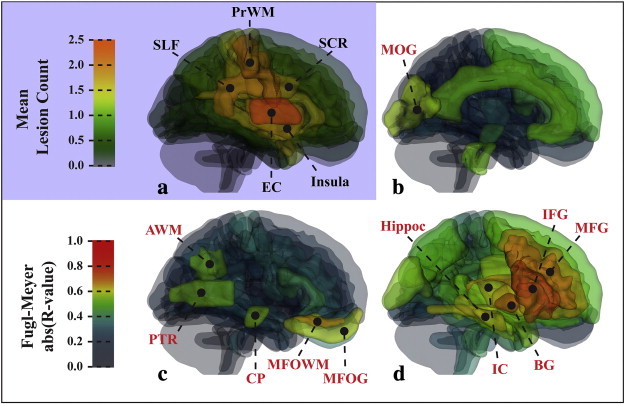
Diagram of ipsilesional ROIs in the control template space, showing the lesion distribution and the correlation coefficients of the regional mean versus composite Fugl-Meyer (FM) score. (a) The lesion distribution of the stroke group was most dense in the EC, SLF, PrWM, SCR, and Insula. (b) Correlations between mean MD and total Fugl-Meyer score were significant (p < 0.05, corrected) in the MOG. (c) Correlations between mean FA and total Fugl-Meyer score were significant (p < 0.05, corrected) in the MFOG, CP, PTR, and AWM. (d) Mean VISC was significantly (p < 0.05, corrected) correlated with Fugl-Meyer score in but not limited to the IFG, MFG, IC, BG, and the IFO. Acronyms: angular gyrus white matter (AWM); basal ganglia (BG); cerebral peduncle (CP); cingulate gyrus (CingG); external capsule (EC); middle occipital gyrus (MOG); middle temporal gyrus (MTG); internal capsule (IC); inferior frontal gyrus (IFG); inferior occipitofrontal fasciculus (IFO); middle frontal gyrus (MFG); middle fronto-orbital gyrus (MFOG); posterior thalamic radiation (PTR); precentral gyrus white matter (PrWM); superior corona radiata (SCR); superior frontal gyrus (SFG); superior longitudinal fasciculus (SLF).

**Fig. 8:**
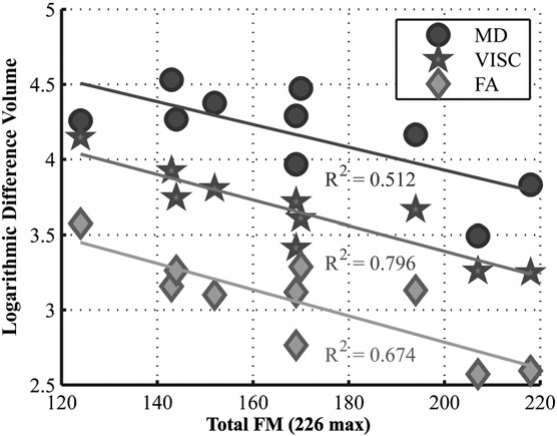
In stroke subjects, the log difference volumes of each DTI metric were correlated with the Fugl-Meyer scores. Log difference volume (LDV) of fractional anisotropy (FA, diamond), mean diffusivity (MD, circle), and voxel-wise indirect structural connectivity (VISC, star) versus Fugl-Meyer (FM) score. The log difference volumes of MD (R^2^ = 0.512), FA (R^2^ = 0.674), and VISC (R^2^ = 0.796), were significantly correlated with FM score. In each subject, the size of the difference volume was smallest in FA and greatest in MD. The difference volume in VISC was greater than in FA and less than in MD.

Voxel-based analyses of VISC, FA and MD were conducted to locate and quantify differences between each stroke survivor and the controls. Each of the three measures identified differences in specific ipsilesional areas of the brain. The VISC revealed ipsilesional regions with lesion-induced changes in structural connectivity, and these differences were not apparent with MD or FA (Fig. 5). Between-group differences (stroke versus control) exclusive to VISC (and not seen with FA or MD) were consistently located outside of the lesion volume (Figs. 5, 6d, 7d). The voxels with significantly different MD (Figs. 6b, 7b) were commonly found inside or near the boundary of the lesion volume (Figs. 6a, 7a), and differences in FA (Figs. 6c, 7c) were largely found in the white matter portion of the lesion volume. In all stroke subjects, VISC detected differences within the lesion volume that were revealed by FA and MD. However, VISC exclusively detected additional voxels affected by the lesion that extended along fiber pathways and within the cortical volume superior to the lesion (Fig. 5). Across all subjects, the number of significantly different voxels between stroke and control groups was always lowest for FA and greatest for MD, with the VISC between the other metrics (Fig. 8).

### 3.5 VISC metric enhances lesion-related differences

**Fig. 9:**
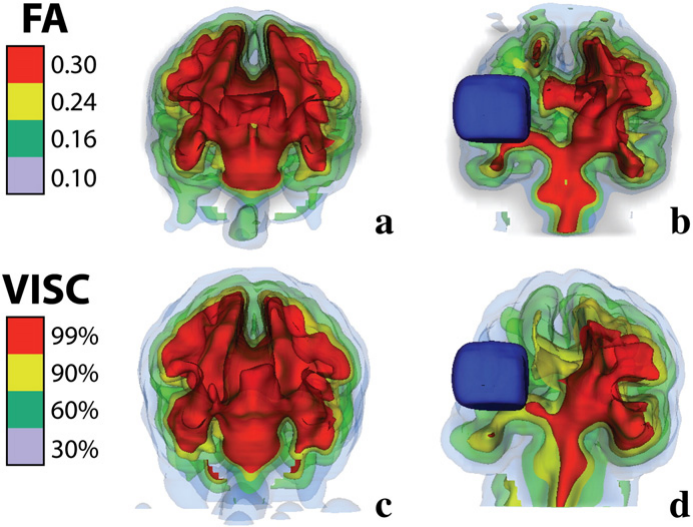
Using 3D isosurfaces, a visual comparison of FA (top) and VISC (bottom) in a control (left) and a stroke (right). The manually-selected lesion volume is shown with a blue surface.

In order to visually compare changes in FA and VISC near the lesion boundary, four isosurfaces were extracted from one stroke subject's FA and VISC images and visualized in 3D (Fig. 9). FA was sensitive to changes local to the lesion, while VISC reflected changes in regions distant from the lesion. VISC effectively thickened white matter regions that were densely connected to surrounding regions, while thinning those regions less connected to surrounding structures. In Fig. 9, the FA showed an asymmetry in the size of the cerebral peduncles, which were not part of the lesion. This asymmetry was enhanced in the VISC image since the paretic cerebral peduncle had a greater decrease in structural connectivity.

### 3.6 Whole-brain LDV and ipsilesional mean of VISC correlates with Fugl-Meyer score

The whole-brain log difference volume (LDV) of each metric in the entire brain was regressed with FM score. In comparison with FA and MD, the LDV of VISC had a greater correlation with Fugl-Meyer score. The relationships between Fugl-Meyer score and each measure were similar and statistically significant (Fig. 8). However, the strength of the association between difference volume and total Fugl-Meyer score was greater for VISC (R^2^ = 0.796, p = 0.0005) than for the MD (R^2^ = 0.512, p = 0.0199) and FA (R^2^ = 0.674, p = 0.0036).

**Fig. 10:**
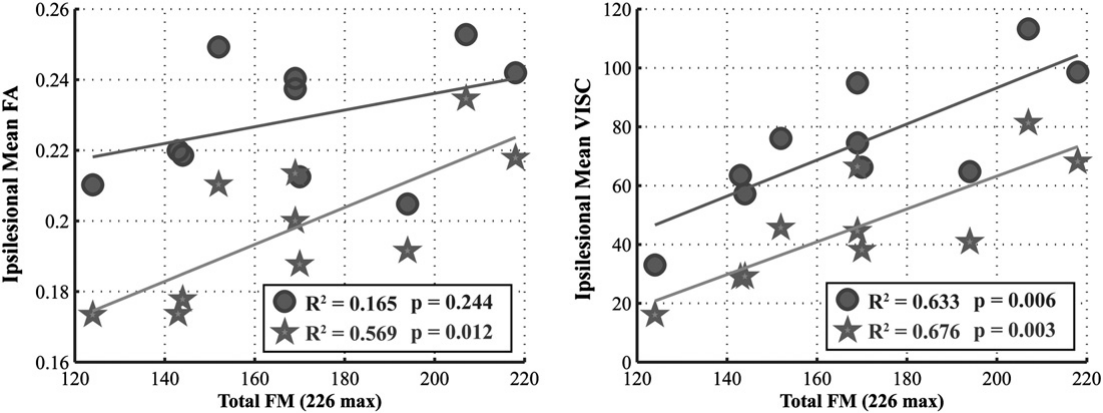
In the stroke group, the mean FA (left) and VISC (right) in the ipsilesional hemisphere were regressed against total FM score. Means were first calculated for only voxels with a nonzero VISC (circle), and then again for all voxels (star). Although the mean FA across ipsilesional voxels with nonzero VISC was poorly correlated (R^2^ = 0.1649, p = 0.2442) with FM score, the mean VISC in these same voxels was significantly correlated (R^2^ = 0.6332, p = 0.0059) with FM. Across all voxels in the ipsilesional hemisphere, the mean FA had a strong correlation (R2 = 0.5691, p = 0.0117) with the behavioral measurements, and mean VISC held its strong correlation (R2 = 0.6763, p = 0.0035).

In the stroke group, the mean FA and VISC in the ipsilesional hemisphere were regressed against total FM score (Fig. 10). Means were first calculated for only voxels with a nonzero VISC, and then again for all voxels. Although the mean FA across ipsilesional voxels with a nonzero VISC was poorly correlated (R^2^ = 0.165, p = 0.2442) with FM score, the mean VISC in these same voxels was significantly correlated (R^2^ = 0.633, p = 0.0059) with FM. Across all voxels in the ipsilesional hemisphere, the mean FA had a strong correlation (R^2^ = 0.570, p = 0.0117) with the behavioral measurements, and mean VISC held its strong correlation (R^2^ = 0.676, p = 0.0035). The mean MD across voxels with nonzero VISC was moderately correlated (R^2^ = 0.304, p = 0.0985) with FM, while this correlation was poor (R^2^ = 0.194, p = 0.2030) for the mean of all ipsilesional voxels.

### 3.7 Association between VISC and upper extremity Fugl-Meyer subscore

**Fig. 11:**
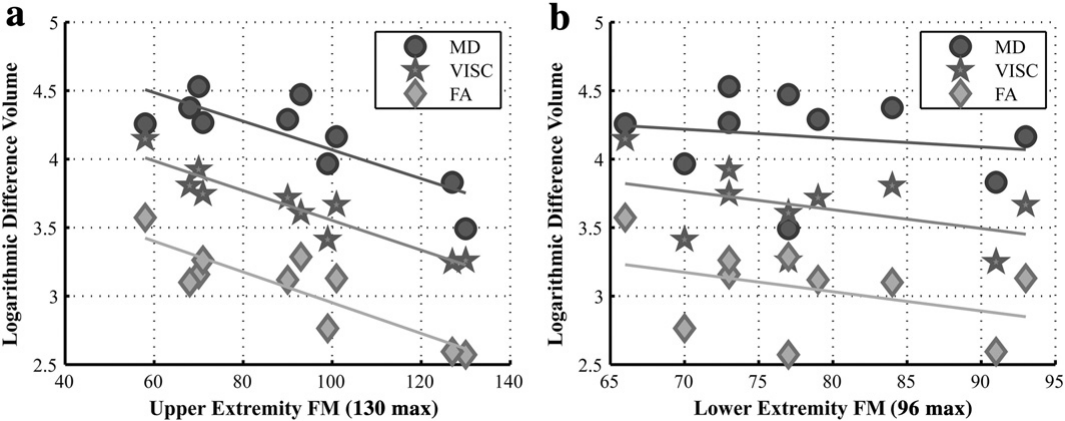
Scatter plots of the log number of significant voxels versus the Fugl-Meyer subscores. (a) The upper extremity FM score was highly correlated with the differences volumes of FA (R^2^ = 0.7453, p < 0.01), MD (R^2^ = 0.6549, p < 0.01), and VISC (R^2^ = 0.8795, p < 0.0001). (b) However, the lower extremity FM score was not correlated with the difference volumes of FA (p = 0.23), MD (p = 0.62), or VISC (p = 0.27).

The first multiple linear regression analysis tested DTI parameter LDVs as predictors of Fugl-Meyer domain scores. No significant (p < 0.05, corrected) associations were detected in this regression. Next, the second multiple regression tested DTI parameter LDV's as predictors of full upper extremity and full lower extremity FM scores. In this case, the LDV of VISC was a significant (R^2^ = 0.821, p < 0.05, corrected) identifier of the upper extremity score (max 130). As shown in Fig. 11 indicate that the upper extremity subscore of the Fugl-Meyer test was strongly associated with LDV for VISC (R^2^ = 0.879, p = 0.00006), but the lower extremity subscore was not significantly correlated with LDV (R^2^ = 0.177, p = 0.226). Furthermore, the simple linear regressions two equivalent tests were repeated with the *mean* DTI parameter across all voxels in the ipsilesional hemisphere as a predictor of the FM subscores. The mean ipsilesional VISC and FA were both significantly (p < 0.05, corrected) correlated with the pain domain score of the Fugl-Meyer. We did not further consider this correlation with pain as a reliable result since eight of the ten stroke subjects scored perfectly (44 out of 44) in the pain domain. No other significant associations were found in these four tests.

### 3.8 *Post-hoc* region-based results

There were significant correlations (p < 0.05, corrected) between the logarithmic difference volume and the total FM score with respect to specific ipsilesional ROIs and metrics (Fig. 6). The correlations were generally strongest with the VISC metric as the predictor in both whole-brain and region-based analyses. The LDV of VISC in the midbrain and association areas (Fig. 6d) was correlated with total FM. The FM score was also correlated with the LDV of MD (Fig. 6b) in the midbrain and LDV of FA in the middle temporal gyrus (Fig. 6c). Table 4 lists the levels of significance in the correlations between the ROI log number of significant voxels and FM score. The mean VISC in the basal ganglia, posterior limb of the internal capsule, and middle and inferior frontal gyri (Fig. 7d) was strongly correlated with the FM score and its upper extremity subscore. Mean MD was correlated with FM score in the middle occipital gyrus (Fig. 7b), and mean FA was significantly correlated with FM score in the fronto-orbital gray and white matter, cerebral peduncle, angular white matter, and posterior thalamic radiation (Fig. 7c). Table 5 lists the levels of significance in the correlations between the ROI mean and FM score.

## 4 Discussion and conclusions

The results of this study suggest that VISC provides unique information about sensorimotor impairment after stroke. VISC was weakly correlated with FA (R^2^ < 0.2) on a voxel-by-voxel basis. Furthermore, the mean VISC and FA across ipsilesional voxels with an FA above 0.15 were correlated with FM score. This suggests that each metric accounts for a different portion of the variance in impaired function in chronic stroke. In both ROI analyses, the VISC metric enhanced a broader set of brain regions. Thus, the VISC metric uniquely identified areas whose structural connectivity may be involved in sensorimotor function after stroke.

Our novel VISC metric differed in chronic stroke survivors compared to controls in brain regions outside the lesion site. Further, the number of voxels with significantly different VISC values correlated with sensorimotor impairment. Although VISC and FA were significantly correlated with Fugl-Meyer, they were weakly correlated with one another. Multiple regression revealed that both the whole-brain log difference volume and ipsilesional mean of VISC provide unique information about upper extremity sensorimotor impairment after stroke. Furthermore, VISC and FA identified different regions with differences between stroke and controls, which further suggests that the VISC provides unique information about the effect of a lesion on brain structure. The ipsilesional mean and log number of significant voxels in the VISC metric both had a greater correlation with sensorimotor impairment than the conventional DTI metrics, MD and FA. This observation may indicate that a broader set of brain areas associated with sensorimotor function can be assessed with the VISC metric as compared to MD and FA. The correlations also seen with MD and FA suggest that local white matter structural diffusion properties near the lesion are associated with higher function. The region-specific correlations exclusive to the VISC metric support that white matter structural connectivity is key to sensorimotor recovery in chronic stroke survivors.

VISC could serve as a unique tool for voxel-wise structural network analysis based on DTI tractography in stroke subjects. Rather than integrating the voxel-wise z-scores of stroke subjects, weighted by a metric derived from region-based structural network analysis (Kuceyeski et al., 2011), or lesion overlap with specific fiber bundles (Riley et al., 2011), the VISC metric is intrinsic and derived directly from DTI tractography. Our conclusion that DTI tractography-based metrics correlate with FM score after stroke is consistent with other studies (Lindenberg et al., 2010; Riley et al., 2011; Zhu et al., 2010).

The VISC metric bears some resemblance to the region-based indirect structural connectivity metric introduced for ROI analysis (Sporns, 2011). This metric is computed for an ROI by summing the product of structural connectivity for all connections through level 2, from all regions, divided by the ROI's number of connections with level 2. In addition to being a voxel-wise metric, VISC is different in that it is the *mean* number of direct (level 1) connections over only indirect neighbors (level 2). Sporns et al. found that region-based direct structural connectivity has a higher correlation with regional resting-state functional connectivity as compared to regional indirect structural connectivity. Nonetheless, we observed that at the voxel level, indirect structural connectivity was more reproducible in the control population than direct structural connectivity. It is possible that since it is a measure of *mean* connectivity, VISC is more robust to voxel-level misregistrations. We incorporated the *α* parameter into VISC so that setting *α* to 0 leads to simply the sum of all direct (level 1) connections to indirect (level 2) neighbors, which is similar to region-based indirect connectivity measures.

Results from the whole-brain analyses revealed the ability of VISC to detect lesion-induced changes in structural connectivity in voxels where FA was not significantly affected. However, the ROI analyses used in this study associated different sets of brain areas with measurements of sensorimotor impairment. Our log difference volume analysis accounted for the mean and variance in the control group at every voxel. This approach identified areas inside of the lesion (as with MD), near lesion boundaries (FA and VISC), and distant integrative association areas (as with VISC). On the other hand, the means of FA and VISC were correlated with FM score in prefrontal areas and the basal ganglia. A change in a regional mean could be caused by a large localized change or a moderate change distributed throughout the region. Due to this uncertainty, the correlations with regional means are difficult to interpret. In combination, the two ROI analyses do not necessarily agree on which regions are associated with sensorimotor impairment after stroke. Inconsistencies between the results of the ROI analyses places limits on the insights gained about the roles of specific brain areas in sensorimotor function after stroke. Whole-brain voxel-based analyses may serve as an objective and more reliable tool for assessing stroke lesions; however, this study was limited by the relatively small number of stroke subjects, and it is possible that the sample size impacted the region-based results.

The voxel-based approach used for the VISC metric in the current study has some advantages over ROI analyses. Voxel-based analysis is not subject to region of interest selection and a priori information. This is important since ROI segmentation may be sensitive to lesion-related changes in structure in stroke survivors. Voxel-wise metrics also provide more sensitive and specific localization of spatial variations, and contrast maps may be visualized in a standardized image space. The VISC metric is also intrinsic and excludes direct neighbors from the average, being based on number of neighbors rather than number of connections. Scheinost et al. introduced a similar intrinsic voxel-wise functional connectivity metric to alleviate the need for a threshold in resting state functional resonance imaging analysis (Scheinost et al., 2012), which produced advantages similar to the voxel-based VISC analysis for DTI data.

Correlations between the upper extremity Fugl-Meyer score and DTI metrics, along with the absence of a correlation between DTI metrics and lower extremity Fugl-Meyer score, could provide insight into the role of white matter structural connectivity in motor impairment. Specifically, correlation analyses between FM subcomponents and VISC volume of significance may suggest that structural connectivity of integrative brain regions may play a larger role in upper extremity motor function than in lower extremity function. The FA of association cortical regions has been previously shown to correlate with upper extremity motor function in stroke subjects with severe impairment. For example, one study found that upper extremity Fugl-Meyer score was correlated with fiber number asymmetry (R = − 0.80; p < 0.001) and regional fractional anisotropy asymmetry (R = − 0.71; p < 0.001) in the corticospinal tract (Lindenberg et al., 2010). Although such past work and this study did not find correlations between DTI measures and lower extremity Fugl-Meyer scores, other measures of lower extremity function may lead to positive results.

Partial volumes of fiber populations in a voxel will affect the calculation of VISC because the metric is based on a union of direct connections via multiple penetrating fibers. Image resolution likely has some effect on VISC, but we suspect that normalizing VISC based on resolution may be sufficient to preserve its intensity at each point. If every voxel theoretically included only one white matter fiber, then the connectivity would be based solely on indirect connections via fiber end points. In this case, the indirect neighbors to any voxel would reside in gray matter, and the VISC would reduce to their average joint volume of connected fibers. Even at high image resolution, voxels would often contain multiple fibers from the same bundle. VISC would still take advantage of this partial volume effect since different fibers from the same bundle can bifurcate and converge. A high percent of voxels contain multiple fiber bundle populations at current resolution. On the other hand, the VISC calculation is likely affected by the diffusion tensor model, which fails to resolve crossing or kissing white matter fiber bundles. This is a limitation of the current study, and it must be considered in the physiological interpretation of VISC.

Our tractography technique suffers from the limitations introduced in any DTI tractography method. The diffusion tensor model assumes that any voxel is populated by white matter fibers with only one fiber orientation. This assumption is not valid in the majority of voxels. A higher-order tensor model would improve the physiological relevance of VISC. Also, we did not account for crossing white matter fiber bundles in our tractography algorithm. If a higher order tensor model were used, then the indirect voxel-based connectivity would need to account for voxels with multiple fiber populations.

Tractography methods and their application to stroke subjects may be criticized because they are correlate with fractional anisotropy. For example, fiber density, calculated as the number of reconstructed fibers that intersect a voxel or region, is correlated with FA (Roberts et al., 2005). We also considered the correlation of VISC and FA for different indirect connectivity weighting factors, *α*. The VISC and FA of voxels with FA above 0.15 were weakly correlated, as shown in Fig. 4b. As the influence of total connection count on VISC was increased by adjusting *α* from 1.0 to 0.0, the correlation of VISC with FA increased until *α* = 0.7 and then decreased. This suggests that the number of indirect connections to a voxel and the average brain volume connected to these indirect neighbors may each provide unique information about local FA.

Another criticism of DTI tractography is the issue of performing tractography in brain images that contain lesions. Although DTI metrics of brain structure based on tractography have been formulated(Buch et al., 2012; Riley et al., 2011) many investigators have avoided performing tractography in the stroke population due to complex changes in diffusion anisotropy associated with the lesion (Budde and Frank, 2010). As an alternative to performing tractography in stroke subjects, one study (Buch et al., 2012) calculated tractography-based voxel-wise statistics and local structural network measures for a control group within a spatially normalized image space, and then each stroke subject's lesion was warped into the standardized space to localize abnormal anatomy. Our results suggest that a voxel-wise metric based on DTI tractography is useful for predicting sensorimotor impairment in stroke subjects. Thus, while tractography-based metrics must be interpreted with caution, they may have value is assessing the implications of a brain lesion.

Key limitations of this study include concerns with the image registration technique, multiple comparisons correction in voxel-wise analysis, and manual lesion selection. Diffeomorphic registration algorithms have been used previously for aligning diffusion tensor images (Beg et al., 2005). An advantage of diffeomorphic registration is that the transform is guaranteed to have a smooth inverse. Thus, applying a forward transform followed by its inverse leads to negligible error. In this study, a noninvertible registration algorithm was used to align the anatomical MR images. An inverse transform was approximated with an error distribution of 0.5622 ± 0.3326 mm. This error could have increased both false positive and false negative voxel-wise t-test outcomes. Diffeomorphic registration is also superior to other techniques in preserving anatomical topology (Beg et al., 2005). We chose a method with less assumptions of topology since stroke lesions may alter the topology of the anatomy. Note that this likely led to higher registration errors in voxels distant from the lesion. Nonetheless, our analysis accounted for the distribution in error due to misregistration at each voxel because the same registration method in this study was used in the control group.

Another minor concern with this study was that the voxel-wise analysis did not account for misregistration errors. Concerns have been raised with voxel-based approaches when used for group comparison because of image misregistration (Bookstein, 2001). There are a number of studies that have addressed registration in voxel-based approaches. Voxel-based morphometry (Ashburner and Friston, 2000) is an attractive technique in that it allows for objective full-brain analysis between populations. This technique reduces effects of registration errors by performing of spatial smoothing prior to voxel-wise Student's T-tests, and then corrects for multiple comparisons by determining significant clusters of spatially connected voxels. Tract-based spatial statistics (TBSS) (Smith et al., 2006) attenuates the effects of misregistration on voxel-based analysis without spatial smoothing. TBSS skeletonizes the mean FA across spatially normalized subjects, and then uses the FA from a voxel near the skeleton in each subject. The VISC metric of the current study may have been less sensitive to misregistration errors because it based on a mean of fiber volumes that, together, represent global white matter structural connectivity of the brain. Being based on the union of multiple fiber pathways, VISC may also be robust against degenerate fibers that contaminate metrics based on fiber counts or mean fiber length. Although VISC can intrinsically detect changes in global white matter structural connectivity at the voxel level, differences in VISC between neighboring voxels should still be interpreted with caution.

This study's third limitation was the manual delineation of the lesion volume. Initially, registering each stroke subject to a control without any correction resulted in distorted morphology near the lesion boundary. Thus, we manually selected a 3D lesion ROI from the structural MR image and then masked the lesion during registration. Few methods have been proposed for automatically segmenting stroke lesions from an anatomical MR image prior to image registration. One technique automatically calculates a lesion mask by thresholding the displacement of the uncorrected deformation map, and then performs registration with lesion masking (Buch et al., 2012). We decided not to use such an approach since some lesions were too small to be detected by the displacement mapping but still led to misregistration near the lesion boundary. Another study optimized local correlations between T1-weighted images in order to register abnormal anatomy with controls (Avants et al., 2008). Although this approach may have provided more consistent registration in stroke subjects, we feel that our method, which accounted for lesion information, was sufficient for the statistical analyses in this study. Overall, we believe that the errors associated with manual lesion selection were minor compared to the advantages it provided in registration.

Voxel-based analyses of structural connectivity in the stroke population can be used to objectively identify brain areas involved in sensorimotor function and may be a useful tool for understanding impairments. The voxel-wise indirect structural connectivity (VISC) measure opens opportunities to investigate the impact of a stroke lesion on extralesional anatomical connectivity. VISC and conventional DTI measures each explain a significant amount of variance in upper extremity motor impairment after stroke. Future investigations will validate VISC with analyses that are based on artificial lesions in healthy control subjects. The sensitivity of VISC to image resolution must also be investigated. Although VISC was not predictive of lower extremity Fugl-Meyer scores, it may associate with other measures of lower extremity function. In order to confirm that this method may be used across a spectrum of lesion sizes and locations, this technique should be performed in a larger sample of stroke subjects. Future studies investigating the percent damage or change in mean value of ROIs should interpret the results with caution in that these widely-used approaches may lead to strikingly different results. Increasing the subject sample size may reduce these inconsistencies. We conclude that our novel VISC metric based on DTI tractography can provide unique information about upper extremity sensorimotor impairment in chronic stroke.

## Figures and Tables

**Table 1:** Description of the Fugl-Meyer scoring system used in this study.

Fugl-Meyer scoring system	Maximum possible score
**Grand total**	**226**
UE + LE motor	100
UE + LE balance	14
UE + LE sensation	24
UE + LE ROM	44
UE + LE pain	44
**UE total**	**130**
UE motor	66
Parachute responses	4
UE light touch	4
UE proprioception	8
UE ROM	24
UE pain	24
**UE motor total**	**66**
UE reflexes	6
UE movements in and out of Synergy	30
Voluntary movements of wrist and hand	24
UE coordination	6
**LE total**	**96**
LE motor	34
Standing balance	8
Sit without support	2
LE light touch	4
LE proprioception	8
LE ROM	20
LE pain	20
**LE Motor total**	**34**
LE reflexes	6
LE movements in and out of synergy	22
LE coordination	6

**Table 2:** Descriptive characteristics of stroke survivors. UE = upper extremity, LE = lower extremity, f = female, m = male. The maximum possible Fugl-Meyer subscores are printed in bold in the bottom row.

						Fugl-Meyer UE + LE score						Fugl-Meyer UE only		Fugl-Meyer LE only	
Subject ID	Sex	Age (years)	Time since stroke (years)	Lesion location	Paretic side	Total	Motor	Balance	Sensation	ROM	Pain	Total	Motor	Total	Motor
S01	F	60	20.4	Cort	R	170	75	11	3	37	44	93	45	77	30
S02	M	53	8.3	Cort	L	143	51	10	7	31	44	70	26	73	25
S04	M	61	5.3	Cort	R	207	87	10	23	43	44	130	66	77	21
S10	F	58	6.1	Cort	L	169	71	10	22	30	36	90	47	79	24
S11	F	53	17.4	Subcort	R	218	92	14	24	44	44	127	61	91	31
S13	M	46	4.4	Subcort	R > L	169	58	10	24	33	44	99	39	70	19
S14	F	52	4.3	Cort	L	152	49	8	24	37	34	68	22	84	27
S15	M	48	8.1	Cort	R	144	35	10	22	33	44	71	18	73	17
S17	F	65	6.2	Cort	L	124	32	8	4	36	44	58	14	66	18
S19	M	55	6.4	Cort	R	194	74	12	24	40	44	101	42	93	32
						**226**	**100**	**14**	**24**	**44**	**44**	**130**	**66**	**96**	**34**

**Table 3:** For each subject, three tractography derived metrics (fiber count, mean fiber length, and VISC) are compared in terms of their correlation (R^2^ value) with fractional anisotropy.

	Fiber count	Fiber length	VISC(1.0)
**C13**	0.131	0.200	0.195
**C17**	0.174	0.217	0.192
**C18**	0.176	0.220	0.204
**C19**	0.134	0.220	0.204
**C20**	0.075	0.166	0.192
**C21**	0.175	0.211	0.201
**C23**	0.120	0.171	0.154
**C26**	0.139	0.225	0.179
**S01**	0.168	0.248	0.206
**S02**	0.208	0.256	0.249
**S04**	0.125	0.199	0.174
**S10**	0.143	0.207	0.186
**S11**	0.166	0.203	0.176
**S13**	0.150	0.222	0.192
**S14**	0.154	0.206	0.193
**S15**	0.142	0.175	0.170
**S17**	0.026	0.072	0.084
**S19**	0.118	0.153	0.158
**Mean**	0.140	0.199	0.184
**Stdev**	0.041	0.041	0.033

**Table 4:** Significance levels of correlations between the ROI mean of each DTI metric and the total Fugl-Meyer (226 maximum). * indicates the first level of significance (corrected p < 0.05). ** indicates the second level of significance (corrected p < 0.01).

VISC			MD			FA		
Mdbrain	0.00155	**	EC	0.01746	*	MTG	0.00003	*
SOG	0.01290	*	Mdbrain	0.02202	*	IFG	0.06732	
SFWM	0.01582	*	SLF	0.02359	*	SFWM	0.07459	
AWM	0.01689	*	RLIC	0.04980	*	STG	0.07861	
IOG	0.03041	*	CingG	0.05828		PrCWM	0.11204	
ACR	0.03494	*	PrCWM	0.05860		STWM	0.11483	
AG	0.03515	*	STWM	0.05942		Fu	0.11843	
STWM	0.04184	*	SFG	0.06571		SFG	0.13267	
RLIC	0.04844	*	PoCWM	0.07137		AWM	0.16955	
GP	0.06572		MFWM	0.08297		SCC	0.17872	
STG	0.06706		Ins	0.08956		IOG	0.19262	

Acronyms: anterior limb of the internal capsule (ALIC), angular gyrus white matter (AWM), caudate nucleus (Caud), cingulum (CGC), cingulate gyrus (CingG), cerebral peduncle (CP), globus pallidus (GP), hippocampus (Hippo), inferior frontal gyrus (IFG), inferior frontal white matter (IFWM), lateral fronto-orbital white matter (LFOWM), middle frontal gyrus (MFG), middle fronto-orbital gyrus (MFOG), middle fronto-orbital white matter (MFOWM), middle frontal white matter (MFWM), medial lemniscus (ML), middle occipital gyrus (MOG), posterior limb of the internal capsule (PLIC), pons (Pons), precentral gyrus (PrCG), posterior thalamic radiation (PTR), putamen (Put), gyrus rectus (RG), superior frontal gyrus (SFG), superior frontal white matter (SFWM), sagittal stratum (SS).

**Table 5:** Significance levels of correlations between the ROI mean of each DTI metric and the total Fugl-Meyer (226 maximum). * indicates the first level of significance (corrected p < 0.05). ** indicates the second level of significance (corrected p < 0.01).

VISC			MD			FA		
IFG	0.00568	**	MOG	0.03746	*	MFOWM	0.01453	*
IFWM	0.00622	**	Pons	0.05387		MFOG	0.02856	*
GP	0.00662	**	CingG	0.05538		CP	0.04144	*
MFWM	0.01019	*	SFG	0.08993		AWM	0.04931	*
MFG	0.01331	*	IFWM	0.10148		PTR	0.04999	*
Caud	0.01511	*	ML	0.10301		IFWM	0.12120	
Put	0.01836	*	CP	0.12125		RG	0.19977	
Hippo	0.02196	*	CGC	0.12518		PrCG	0.20301	
PLIC	0.02262	*	MFOWM	0.13135		LFOWM	0.20901	
SS	0.02323	*	SFWM	0.13771		MFWM	0.22168	
ALIC	0.02412	*	PLIC	0.21877		ML	0.24035	

Acronyms: anterior corona radiata (ACR), angular gyrus (AG), angular gyrus white matter (AWM), cingulate gyrus (CingG), external capsule (EC), fusiform gyrus (Fu), globus pallidus (GP), inferior frontal gyrus (IFG), insular cortex (Ins), inferior occipital gyrus (IOG), midbrain (mdbrain), middle frontal white matter (MFWM), middle temporal gyrus (MTG), postcentral white matter (PoCWM), precentral white matter (PrCWM), retrolenticular part of the internal capsule (RLIC), splenium of the corpus callosum (SCC), superior frontal gyrus (SFG), superior frontal white matter (SFWM), superior longitudinal fasciculus (SLF), superior occipital gyrus (SOG), superior temporal gyrus (STG), superior temporal white matter (STWM).

## Appendix A Voxel-wise indirect structural connectivity derivation

In this study, we defined a voxel by the position of its center rather than its vertices. In the Digital Imaging and Communications in Medicine (DICOM) standard, the Image Position (Patient) header element with tag (0020,0032) defines the x, y, z coordinates of both the upper left hand corner of the image and the center of the first voxel. Since the DICOM standard defines a voxel by its center in the patient coordinate system, we elected to use this convention in our software and nomenclature.

As a foundation for deriving a voxel-wise connectivity metric, the reconstructed fiber trajectories were expressed as sets of coordinates in the template control subject's DTI image-space with the following mathematical formulation. An image here has *n*_z_ slices, each with *n*_y_ rows and *n*_x_ columns, giving a total of *N* voxels.

Let each reconstructed fiber be represented as a matrix, **F**, of 3D fiber trajectory coordinates, where

(A1) $$F\begin{array}{cc} =\left[\begin{array}{ccccc} f_{1,x} & f_{2,x} & \ldots & f_{M-1,x} & f_{M,x}\\ f_{1,y} & f_{2,y} & \ldots & f_{M-1,y} & f_{M,y}\\ f_{1,z} & f_{2,z} & \ldots & f_{M-1,z} & f_{M,z} \end{array}\right] & f_{j,d}\in R \end{array}\text{.}$$

*M* is the number of points in the reconstructed fiber and *f*_*j*,*d*_ is the *d*th dimension continuous coordinate of the *j*th point along the trajectory. Using nearest neighbor interpolation, the 3D continuous coordinates from **F** are converted into 1D voxel indices. Let these indices be represented in vector $\tilde{f}$, with

(A2) $$\begin{array}{cc} {\tilde{f}}_{j}=\left\lfloor f_{j,x}+0.5\right\rfloor +n_{x}\left\lfloor f_{j,y}+0.5\right\rfloor +n_{x}n_{y}\left\lfloor f_{j,z}+0.5\right\rfloor . & j\in \left[1,2,\ldots ,M-1,M\right] \end{array}\text{.}$$

Next, we represent the set of voxels penetrated by fiber **F** as an inclusion mask over the entire image. Let vector **p** of length *N* (# of voxels) be the voxel-wise inclusion mask of *one* fiber, where

(A3) $$p_{i}=\left\{\begin{array}{cc} 1 & \exists j:{\tilde{f}}_{j}=i\\ 0 & \mathrm{otherwise}\text{.} \end{array}\right)$$

If *p*_*i*_ = 1, this indicates that fiber **F** penetrates the *i*th voxel. Note that all voxels included in **p** are described here as being structurally connected. An *N* × *N* connectivity matrix, $\tilde{X}$, is used to represent the direct connections between the voxels masked by **p** with

(A4) $$\tilde{X}=\left(p^{T}p\right)-\mathrm{diag}\left(p\right)\text{.}$$

Note that

$$\mathrm{diag}\left(p\right)=\left[\begin{array}{ccc} p_{1} & & 0\\ & \ddots & \\ 0 & & p_{N} \end{array}\right]\text{.}$$

If ${\tilde{X}}_{k}$ is the connectivity matrix for the *k*th fiber, then let **X** be the union of connectivity matrices across all *Q* fibers, where

(A5) $$X=\overset{Q}{\underset{k=1}{\cup }}{\tilde{X}}_{k}\text{.}$$

If *x*_*ij*_, an element of **X**, is 1, then the *i*th and *j*th voxels are penetrated by least one common fiber. Thus, **X** is a voxel-wise direct connectivity matrix. Next, if the *i*th and *j*th voxels share at least one common direct neighbor but are not directly connected by a fiber, then we define them as indirect neighbors. Let matrix **Y** be the voxel-wise indirect structural connectivity (VISC) matrix, where

(A6) $$y_{\mathrm{ij}}=\left\{\begin{array}{cc} \left(1-x_{\mathrm{ij}}\right)\left(1-\prod_{k=1}^{N}\left(1-x_{\mathrm{ik}}x_{\mathrm{kj}}\right)\right) & i\neq j\\ 0 & \mathrm{otherwise}\text{.} \end{array}\right)$$

Note that term (1 − *x*_*ij*_) excludes voxels that are direct neighbors, and term (1 − ∏_*k* = 1_^*N*^(1 − *x*_*ik*_*x*_*kj*_)) includes pairs of voxels that *share* at least one direct neighbor. Finally, the VISC index for the *i*th voxel is

(1) $${\mathrm{VISC}}_{i}=\frac{y_{\left(i\right)}X1}{y_{\left(i\right)}1}=\frac{\sum_{j}^{N}\left(y_{\mathrm{ij}}\sum_{k}^{N}x_{\mathrm{jk}}\right)}{\sum_{j}^{N}y_{\mathrm{ij}}}$$

where **1** is the summation vector of length *N* and **y**_(*i*)_ is the *i*th row vector from matrix **Y**.

Note that the denominator of VISC is the total number of voxels indirectly connected to the *i*th voxel, whereas the numerator is the summation of direct connections to these same voxels. The nested summation in the numerator of Eq. (1) is over *x*_*jk*_, which gives the total number of *direct* connections to the *j*th voxel. The *y*_*ij*_ terms are inclusive to only *indirect* connections to the *i*th voxel.

Previously introduced voxel-wise metrics based on DTI tractography, such as fiber count and mean fiber length (Roberts et al., 2005), are correlated with FA. In order to consider whether fiber count information affected the correlation of VISC with FA, we incorporated a connection count weighting factor *α*. This contrast mechanism gives weight to the total number of connections to a voxel through structural connectivity level 2 (Sporns, 2011) neighbors. As *α* is decreased from 1 to 0, the VISC approximates the *total number* rather than the *mean number* of direct connections to a voxel's indirect neighbors, with VISC parameterized by *α* as

(2) $$\mathrm{VISC}{\left(\alpha \right)}_{i}=\frac{y_{\left(i\right)}X1}{{\left(y_{\left(i\right)}1\right)}^{\alpha }}.$$
